# Visual–tactile shape perception in Argus II Participants: The impact of prolonged device use and blindness on performance

**DOI:** 10.1167/jov.25.12.19

**Published:** 2025-10-14

**Authors:** Stephanie Saltzmann, Noelle Stiles

**Affiliations:** 1 Department of Neurology, Robert Wood Johnson Medical School, Rutgers University, New Brunswick, NJ, USA; 2 Center for Advanced Human Brain Imaging Research, Brain Health Institute, Rutgers University, New Brunswick, NJ, USA; 3 Department of Ophthalmology, Keck School of Medicine, University of Southern California, Los Angeles, CA, USA

**Keywords:** retinal prostheses, shape perception, perceptual learning, blindness, low vision

## Abstract

In Stiles et al. (2022), we showed that experienced Argus II retinal prosthesis users could accurately match visual and tactile shape stimuli (*n* = 6; ≤42 months of use). In this follow-up paper, we studied longer using participants (*n* = 5; ≤121 months of use) to evaluate visual and multisensory performance over prolonged visual restoration. With the combined cohort of participants from both studies (*N* = 11), we found that there was a significant positive correlation in multisensory performance up to the median duration of use (42 months) and a positive slope fit but not a significant correlation for the median duration of use and beyond. Therefore, there seems to be evidence for initial performance improvement with Argus II use. Nevertheless, there is also evidence for substantial individual differences with more extended device use, supported by a participant self-evaluation/questionnaire. Variations in the frequency of device usage, device functionality, or neurostructural plasticity could contribute to these individual differences. We also found a negative correlation in Argus II participants (*N* = 11) between task performance and the duration of blindness, potentially indicating the deleterious effects of atrophy and neurostructural changes during blindness on visual restoration functionality. Finally, a *d*′ analysis showed that the Argus II participants in all tasks (including tactile–tactile matching) had significant differences in sensitivity and bias relative to controls, highlighting variation in the shape task strategy. Overall, these data highlight individual differences in performance over prolonged device use and the negative impact of prolonged blindness on visual restoration.

## Introduction

Retinal prostheses restore visual perception by electrically stimulating surviving retinal ganglion and bipolar cells following retinal degeneration. The first Argus II retinal prosthesis device (Second Sight Medical Products, Sylmar, CA) was implanted in the United States in 2007 (McDonald, 2019), the Alpha IMS device (Retina Implant AG, Reutlingen, Germany) was first implanted in 2010 (Stingl et al., 2013), and the Prima bionic vision system (Pixium Vision, Paris, France) was first activated in 2018 (Pixium Vision, 2018). Because clinical trials and device commercialization have been relatively recent, research studies have primarily focused on the visual performance of participants in the first few months and years following implantation. The device typically functions optimally at this early time point, and the participant uses the device frequently. In general, the most extended duration of prosthesis use examined in the existing literature seldom exceeds 5 years (Ahuja et al., 2011; Barry & Dagnelie, 2016; Cosendai, Arsiero, Patnode, Greenberg, & Argus II Study Group, 2011; da Cruz et al., 2013; Dagnelie et al., 2017; Dorn et al., 2013; Duncan et al., 2017; He, Huang, Caspi, Roy, & Montezuma, 2019; Horsager, Greenberg, & Fine, 2010; Kotecha, Zhong, Stewart, & da Cruz, 2014). To date, there are only a few studies involving participants with more than 9 years of device use (Caspi et al., 2018; Christie et al., 2023; Yücel et al., 2022). Therefore, device functionality over longer durations of use (such as over 5 years) is still unclear for general retinal prostheses, particularly the Argus II device. This study aimed to evaluate multisensory and visual function in Argus II participants ranging from 10 months (less than a year) of use up to 121 months (10 years). The novelty of our study lies in both the broad time range our participants have been utilizing the Argus II device and our explicit investigation into how the extended duration of use impacts both multisensory and visual function.

This study built on our previous paper, which was the first, to our knowledge, to investigate the integration of artificial visual and natural tactile shape information (Stiles, Weiland, & Patel, 2022). The primary aim of the previous paper was to evaluate the visual and tactile information integration among Argus II participants and to determine their unisensory and multisensory learning trajectories within the first few years of device use. We found that the Argus II participants could match two shapes using artificial vision alone (visual–visual) or somatosensation alone (tactile–tactile) on average above chance (50%). In addition, we found that multisensory matching (visual–tactile) was significantly correlated with the duration of device use (up to 42 months of device use). The longer-using participants also performed multisensory matching well above chance. This previous publication included two control groups: a sighted control group with normal vision (*n* = 10) and a sighted control group with simulated low vision (*n* = 8). The control groups performed the visual–visual and visual–tactile shape matching significantly more accurately than the Argus II participants. Still, the tactile–tactile shape matching was not significantly different between the groups.

Our previous study evaluated shape perception in Argus II participants (*n* = 6) with approximately 1 to 3.5 years (10–42 months) of device use (Stiles et al., 2022). In this study, we extended that investigation to compare those participants to a new cohort of participants (*n* = 5) with approximately 3.5 to 10 years (44.5–121 months) of device use. This new combined analysis (*N* = 11) permits a more robust evaluation of how visual and multisensory shape-matching performance evolves over time (or device use). We also asked participants to self-evaluate their change in prosthesis functionality over time, which bolsters our correlation analyses. In addition, this larger cohort of participants (*N* = 11) relative to our previous paper (*n* = 6) provides a higher statistical power to key correlations between Argus II functionality and participant demographics, such as duration of blindness and participant age. Finally, the larger total participant cohort also permits a novel evaluation of response bias (signal detection analysis) during the shape-matching task, which we hypothesize will find differences in task strategy between the Argus II participants and sighted controls.

Due to the limited number of multisensory studies on participants with retinal prostheses, a signal detection analysis has not been previously performed on this type of participant functionality (multisensory shape perception). This study used *d*′ and response bias (*C*) as signal detection measures to evaluate differences between our sighted control groups and our Argus II participant group, thereby highlighting variations in participants’ decision-making processes and approaches to visual and tactile tasks. Response bias can be used as a measure of a participant's tendency to respond “same” or “different,” whereas *d*′ is used to evaluate a participant's ability to discriminate signal and noise (Green & Swets, 1966; Macmillan & Creelman, 2005; Swets, 1961; Swets, Tanner, & Birdsall, 1961; Tanner & Swets, 1954). We evaluated both measures in the entire cohort of Argus II participants to study these factors in their performance.

Overall, this study investigated the impact of prolonged retinal prosthesis device usage on performance in a visual and multisensory shape-matching task. We used correlation analyses to study the effects of duration of device usage, duration of blindness, and age on performance. We also performed a signal detection analysis to evaluate task strategy relative to sighted control groups.

## Methods

### Participants

Ten participants blinded by retinitis pigmentosa and one blinded by Batten disease with implanted Argus II retinal prostheses participated in this study (two females, nine males; mean Argus II participant age, 62.36 ± 12.13 years; range, 44–76 years) (Table 1). All Argus II participants had light perception or less in both eyes (Table 1) and wore an eyepatch during the study if they reported natural light perception. The Argus II participants had an average of 33.18 years of blindness and an average of 53.86 months since Argus II device implantation (duration of prosthesis use) (Table 1). The Argus II device function, training, and frequency of use are detailed below in the Argus II retinal prosthesis device section. Two Argus II participants previously had an Argus I implanted in their other eye before receiving the Argus II implant. For these two participants, the duration of the Argus I use was counted within the period of blindness due to the significantly lower resolution of the Argus I device (16 electrodes total).

**Table 1.** Argus II participant demographics. The age, gender, duration of blindness, and duration with the Argus II device are detailed. The participant's level of visual perception (left and right eye) is listed as light perception (LP) or no light perception (No LP). If an eye patch was used during the experiment to block natural vision, then a “yes” is listed under the columns for each eye patch column for the left and/or right eye.

Subject ID	Age (y)	Gender	Blindness duration (y)	Argus II duration (mo)	Vision, left eye	Vision, right eye	Eye patch, left	Eye patch, right
A1	61	F	16	42	LP	No LP	Yes	No
A2	69	M	24	30	LP	LP	Yes	Yes
A3	46	M	20	19.5	LP	LP	Yes	Yes
A4	65	M	26	23	LP	No LP	Yes	Yes
A5	75	F	52	10	No LP	No LP	No	No
A6	76	M	38	19	No LP	No LP	Yes	Yes
A7	44	M	23	121	LP	LP	Yes	Yes
A8	62	M	39	99.5	LP	LP	Yes	Yes
A9	76	M	56	112	LP	No LP	Yes	Yes
A10	46	M	25	72	No LP	LP	Yes	Yes
A11	66	M	46	44.5	No LP	No LP	Yes	Yes

Ten age-matched sighted participants performed the experiments (seven females, three males; mean sighted participant age, 63.5 ± 4.70 years; range, 55–69 years) (Supplementary Table S1). The sighted controls used their natural visual and tactile perception to perform the tasks. The experiment with the sighted controls was performed using the same protocol and methods as the Argus II participants.

Eight sighted participants with simulated ultra-low vision also performed the experiments (five females, three males; mean sighted participant age, 41.88 ± 15.48 years; range, 25–68 years). The age, visual acuity of the right eye (eye patch on the left eye), and the visual acuity of the right eye with simulated ultra-low vision are reported in Supplementary Table S2. These sighted controls’ right eye visual acuity ranged from 20/20 to 20/80+4 (Supplementary Table S2). The right eye visual acuity with simulated ultra-low vision ranged between 20/600 and 20/1000–2 with a mean of 20/775+2 (Supplementary Table S2). The procedure for measuring visual acuity and the simulation of ultra-low vision are detailed in the Simulation of ultra-low vision with sighted controls section, below.

All participants gave written informed consent, and all experiments were approved by the University of Southern California Institutional Review Board. This research adhered to the tenets of the Declaration of Helsinki.

### Argus II retinal prosthesis device

The Argus II retinal prosthesis offers visual perception to individuals affected by retinitis pigmentosa (and related diseases) by stimulating the remaining retinal ganglion cells using an epiretinal microelectrode array (Humayun et al., 2012; Luo & da Cruz, 2014; Luo & da Cruz, 2016; Weiland, Cho, & Humayun, 2011; Zhou, Dorn, & Greenberg, 2013; Zrenner, 2013). The Argus II is no longer in production, and Second Sight Medical Services has merged with Nano Precision Medical to form Cortigent.

**Figure 1.**
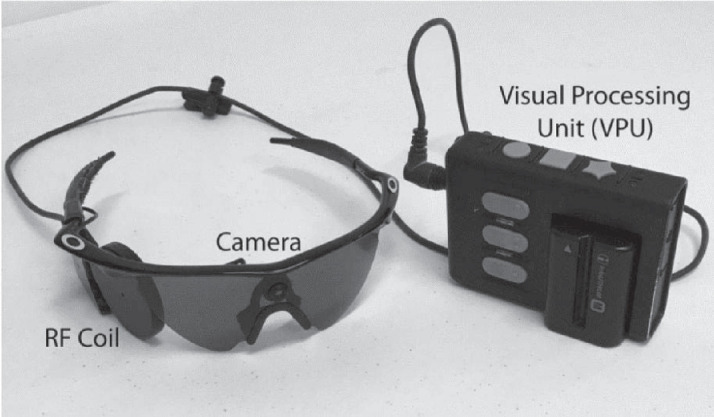
Image of the external components of the Argus II retinal prosthesis system (details in the Methods section).

This array connects to a scleral buckle, housing a programmable stimulator and coil, which wirelessly receives data and power from a radiofrequency (RF) coil integrated into a pair of glasses (see Figure 1). The device incorporates a small camera positioned on the bridge of a pair of sunglasses to capture the visual environment. The visual feed from the camera is transmitted via wire to a vision processing unit (VPU) worn on a belt, which processes the visual data and sends stimulation parameters back through the wire to the glasses-mounted RF coil. The Argus II device provides a resolution of 6 × 10 pixels, displayed across an 11° × 18° field of view (He et al., 2019). Visual perception varies greatly among users, as they do not regain normal vision but instead experience a form of artificial vision made up of patterns of light and dark (phosphenes). Users may experience flashes of light, low-resolution shapes and motion, contrast-based vision, and black-and-white vision (Caspi, Dorn, McClure, Humayun, & Greenberg, 2011; da Cruz et al., 2013; Dorn et al., 2013; Stronks & Dagnelie, 2014). Notably, in Argus II users, visual perception is learning dependent and requires training to interpret these visual patterns.

Approximately 1 month after surgical implantation of the device, Argus II users revisit the medical center for device calibration and to report their initial experience with the device. Following this, users are granted permission to take the glasses and VPU home for personal use. Users could have participated in rehabilitation training offered by Second Sight, typically spanning 3 to 4 hours per day over multiple days. Both visual and tactile training are incorporated into rehabilitation. Second Sight offered rehabilitation training that involved, but was not limited to, mobility and orientation training, the integration of tactile feedback, hand–eye coordination, and contrast sensitivity. Seven of the 11 Argus II participants involved in this study underwent rehabilitation training. The frequency of device usage among the Argus II participants ranged from once daily to once a month. Further details regarding the rehabilitation training and individual participant device usage frequencies are provided in Supplementary Table S3.

### Experimental setup

The materials and methods in the current study are identical to that of Stiles et al. (2022). The experiment occurred within a designated experimental space at the Keck School of Medicine, University of Southern California. A table covered with black felt was placed against a large, blocked window or wall, which was also draped with black felt fabric. Lighting within the experimental room was provided by internal artificial lighting and natural light coming in through an adjacent open window. Video recording of the experiment was conducted to facilitate supplementary data analysis and to capture video demonstrations of the tasks (with participant consent).

The Argus II participants used their retinal prosthesis to view white shapes on the black felt fabric. At times, participants using the Argus II prosthesis encountered challenges due to their height, making it difficult to observe the shapes on the table while seated without assuming an oblique angle. In such instances, they could stand at the table's edge, affording them a less oblique view to observe the shapes from above. However, most trials were performed in a sitting position.

### Experimental stimuli

**Figure 2.**
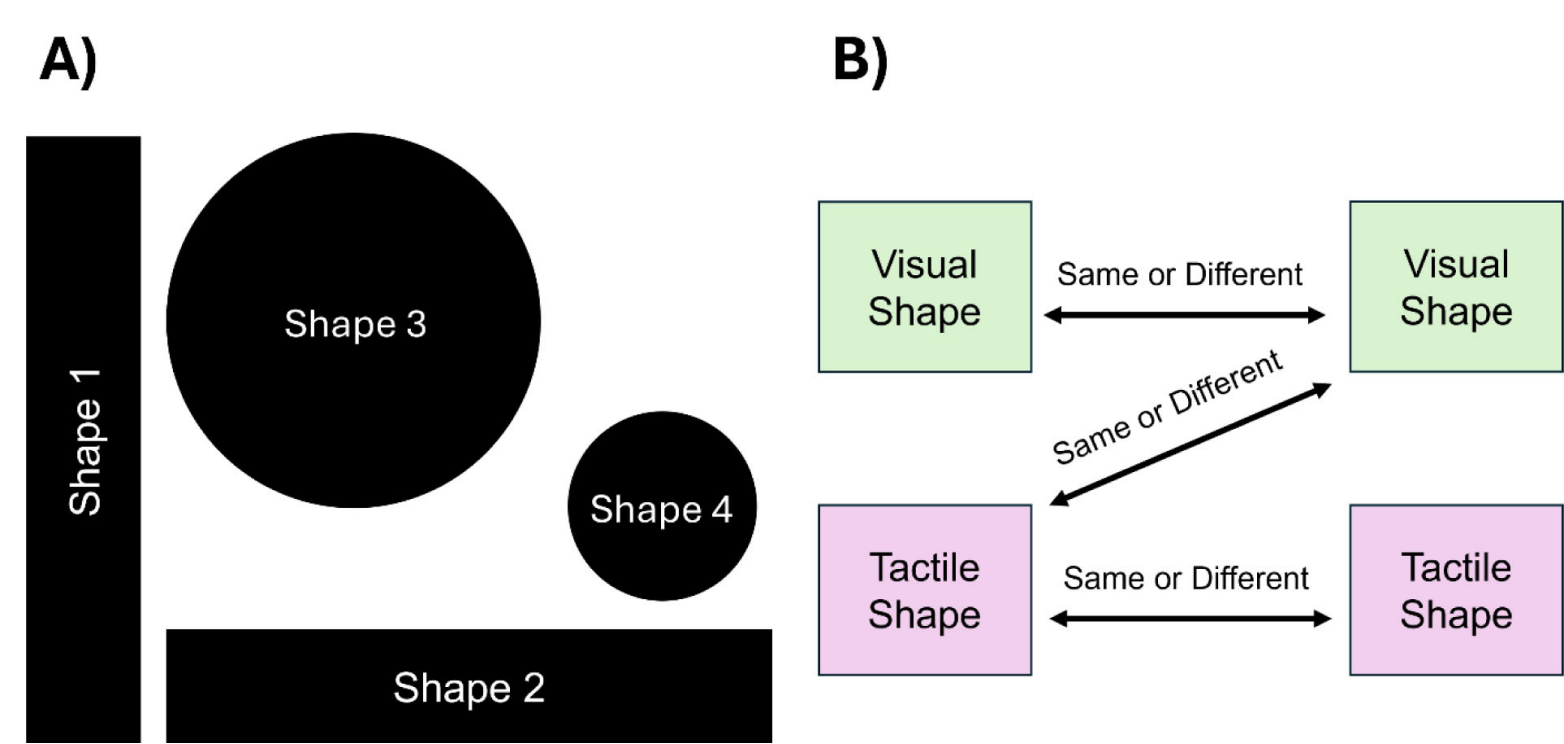
(**A**) The shapes used in the shape-matching tasks. (**B**) A schematic depicting the three types of object shape comparisons performed in the shape-matching tasks.

Four shapes were selected for the tactile and visual components of the experiment (Figure 2 and Supplementary Figure S1) (Stiles et al., 2022). These shapes were cut from white poster boards, giving each a thickness of approximately one-quarter of an inch. Shape 1 was a vertical rectangle measuring 8 inches in height by 1.5 inches in width; shape 2 was a horizontal rectangle measuring 1.5 inches in height by 8 inches in width; shape 3 featured a large circle with a diameter of 5.5 inches; and shape 4 was a small circle with a diameter of 1.5 inches. Each shape was marked with its corresponding shape number (shapes 1–4) on the backside which matched the stimulus numbers pre-recorded in the experimental notebook in the order of their presentation during the task. Shapes 1 and 2 constituted a single physical item or shape, with shape 2 being oriented horizontally and shape 1 vertically during the presentation of stimuli.

### Experimental preparation and stimuli randomization

An experimental notebook recorded participants’ responses throughout the experimental tasks. This notebook was prepared in advance, and the randomization of stimulus order was performed using the *randperm* function in MATLAB (MathWorks, Natick, MA) for each block of experimental trials (tactile–tactile matching block, visual–tactile matching block, and visual–visual matching block). Within each block, stimuli were presented in pairs, encompassing all possible shape pairings repeated once, with each shape paired with itself repeated once more. The exception was the exclusion of the pairing between shape 2 (horizontal rectangle) and shape 3 (large circle) due to redundancy. Consequently, the total number of trials per block amounted to 18. In other words, participants considered four shapes paired with four others, generating 16 combinations, subtracting two redundant pairings (for shape 2 and shape 3), and adding four extra same-shape pairings (e.g., shape 2 vs. shape 2). With three experimental blocks and 18 trials per block, the total number of trials conducted for the experiment equated to 54.

### Overall experimental task

**Figure 3.**
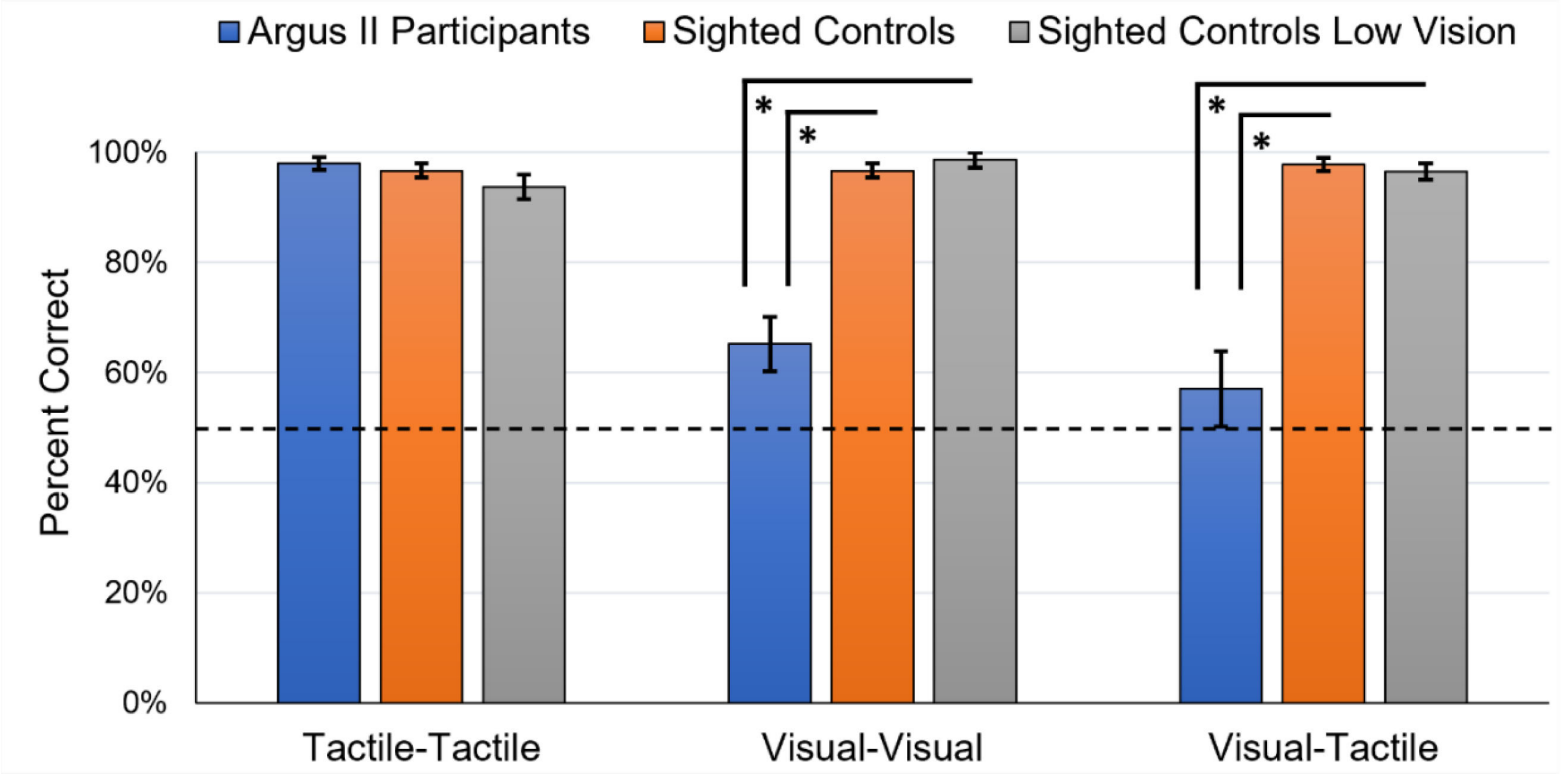
Shape-matching performance. Average percent correct for the shape-matching tasks for the Argus II participant group compared with the sighted participant group and the sighted participant group with simulated ultra-low vision. The dashed line represents chance (two-alternative forced choice [2AFC], or 0.50 fraction correct). Error bars represent standard error.

The experiment was comprised of three blocks, each consisting of 18 trials. Sequentially, the first block entailed the tactile–tactile matching task, followed by the visual–tactile matching task in the second block, concluding with the visual–visual matching task in the third block (see Figure 3B). This order of experimental blocks was deliberately chosen to minimize the potential transfer of visual shape learning across blocks. During the visual–tactile matching block, participants engaged in a touch-to-vision matching task, wherein the tactile stimulus was consistently presented before the visual stimulus. Participants were allowed brief intermissions between the experimental blocks to ensure optimal comfort and attention. Throughout the experiment, participants received no feedback regarding their performance. The experimental design involved presenting each shape individually, prompting participants to decide whether the subsequent shape matched or differed from the preceding one.

### Tactile–tactile shape-matching block: Experimental task details

During the tactile–tactile matching task, Argus II participants deactivated their Argus II device. Throughout this task, Argus II participants, sighted controls, and sighted controls with simulated ultra-low vision relied on their innate tactile perception. Participants were seated at a table covered with two layers of black felt—one to conceal the table surface and another to conceal the shapes placed underneath for tactile exploration. Positioned on the participant's left side, the experimenter initiated each trial by concealing all shapes under one layer of black felt on the table. The experimenter then retrieved one shape and moved it beneath the black felt fabric, positioning it in front of the participant. Participants were instructed to explore the concealed shape by placing their hands on top of the fabric and feeling for the shape. After this exploration, the experimenter replaced the first shape with another concealed shape beneath the black felt fabric. Participants were then tasked with exploring this second shape through touch and determining whether it matched the first shape or differed in size, shape, or orientation. Throughout the task, participants were reminded of the criteria for deciding the similarities or differences between shapes. Upon examining the second shape, participants verbally indicated whether or not it matched the preceding one. The experimenter recorded participants’ responses in the designated notebook.

### Visual–tactile shape-matching block: Experimental task details

During the visual–tactile shape-matching task, Argus II participants utilized their Argus II device for artificial vision and their natural tactile perception. In contrast, sighted participants with and without simulated ultra-low vision relied on their natural tactile perception and simulated ultra-low vision or natural visual perception (respectively) for this task. Participants and experimenters were seated at a table covered with black felt, mirroring the setup used for the tactile–tactile matching block. The experimenter initiated the visual–tactile shape-matching task by sliding a shape beneath the black felt fabric and presenting it to the participant for tactile exploration. After the participant concluded their tactile examination on the surface of the black felt fabric, the initial shape was moved away from the participant beneath the fabric. Next, the experimenter introduced the second shape by placing it on top of the black felt fabric directly in front of the participant, contrasting a white object against the black fabric background. The participants were instructed to view the shape but not to touch it. Upon visual exploration, the participant was prompted to determine whether the second shape matched or differed from the first shape based on size, shape, or orientation. Following visual examination, participants verbally indicated their assessment of the shape as either the same or different from the initial one. The experimenter recorded participants’ responses in the designated notebook and returned the shape to beneath the black felt fabric.

### Visual–visual shape-matching block: Experimental task details

During the visual–visual matching task, Argus II participants relied on their Argus II device for artificial vision, whereas sighted participants used their natural visual perception. Additionally, sighted participants with simulated ultra-low vision utilized the simulated ultra-low vision for this task. At the outset of the task and between trials, all shapes were concealed beneath a layer of black felt fabric in front of the experimenter. The task began when the experimenter retrieved the first shape from under the black felt fabric and placed it in front of the participant on the surface of the black felt fabric. Participants visually inspected the shape without touching it. Subsequently, the experimenter removed the shape and returned it to beneath the black felt fabric. Next, the experimenter retrieved a second shape from beneath the black felt fabric and presented it in front of the participant for comparison with the first shape. Participants then determined whether the second shape matched or differed from the first based on size, shape, or orientation. Participants verbally reported their assessment of the shape, and the experimenter recorded their responses.

### Simulation of ultra-low vision with sighted controls

The ultra-low-vision simulation involved using a monocular eye patch and an opaque face mask with a small viewing window (Stiles et al., 2022). This opaque face mask was crafted by covering a clear face shield with black duct tape, rendering the interior of the shield entirely black except for a window measuring 0.28 × 0.435 inches. The dimensions of the viewing window were calculated to provide an approximate visual field of view of 11° × 18°, with the viewing window positioned approximately 1.40 inches from the right eye. (These numbers are approximate, as individual subjects may have slightly different eye and head geometries relative to the mask; therefore, there may be minor variations in the actual visual field experienced by each participant.) Before black duct tape was applied to the surface of the mask, four Bangerter occlusion foils were affixed over the region of the right eye window. These foils, designed to simulate low vision, included filters with blur levels corresponding to visual acuities of 20/70, 20/200, 20/200, and 20/100. During the experiment, participants first put on a monocular eye patch over their left eye, followed by the opaque face mask. The small window in the opaque face mask was aligned with the participant's right eye. To maintain consistency in the field of view through the window, participants were instructed not to adjust the position of the mask closer to their right eye after it had been properly positioned on their head.

Evaluation of participants’ right eye visual acuity, with and without the opaque face mask, was conducted using a low-vision letter chart featuring Sloan letters in logMAR increments from Precision Vision (Woodstock, IL). Visual acuity for the right eye was assessed with a monocular eye patch worn over the left eye (Supplementary Table S2).

Sighted controls with simulated ultra-low vision engaged in the shape-matching tasks following the same protocol as the Argus II participants and the other normally sighted controls. During the tactile–tactile matching task, participants were allowed to remove the mask and eye patch, given the purely tactile nature of the task. However, for the visual–tactile and visual–visual matching tasks, participants were required to wear both the opaque face mask and the monocular eye patch.

### Post-experiment survey

The Argus II participants were contacted after completion of the experiment and asked about their perceptual experience since receiving the Argus II retinal prosthesis. Specifically, the participants were asked to select one of five responses about their capabilities with the Argus II device. The options were as follows: (a) remained the same functionally since receiving the device; (b) continued to improve in functionality consistently over time; (c) continued to become worse in functionality consistently over time; (d) improved initially after activation, peaked in functionality, and since declined in functionality; or (e) became worse initially after activation, reached a low point in functionality, and since improved in functionality. Out of 11 Argus II participants, we received responses from 10. (Note: This follow-up question was asked at the visit or shortly afterward for the five new participants; see duration of use in Table 1). For participants from the original cohort, the question was asked from 2023 to 2024, whereas the original experiment was ∼5 years prior to this. If a participant had stopped using the device, we asked them to estimate their performance over their time of usage. Therefore, for the original six participants, their duration of use was about 5 years at the time of the question, plus their original duration of use listed in Table 1. Overall, the 10 participants ranged from approximately 44.5 to 121 months of use when they answered this question, with an average of 86.5 months.

### Statistical analyses

All statistical tests were performed with SPSS Statistics software (IBM, Chicago, IL) and were based on a priori hypotheses. To test the a priori hypothesis that the mean accuracy significantly varied among participant groups for each shape task (tactile–tactile, visual–visual, and visual–tactile tasks), one-way analyses of variance (ANOVAs) were conducted. One-way ANOVAs for each shape task minimized the total number of comparisons by assessing performance across groups for each task individually (i.e., Argus II participants, sighted controls, and sighted controls with simulated ultra-low vision) rather than comparing across tasks. In instances where the groups exhibited significant differences according to the one-way ANOVA, subsequent statistical tests (with a Bonferroni multiple comparisons correction) were conducted to examine pairwise comparisons among the participant groups. The correlation analyses were performed using Pearson's correlation. All ANOVAs and correlations were conducted using both traditional hypothesis testing and Bayesian analyses. Bayes factors (BFs) that favor the alternative hypothesis are represented as BF_10_, whereas values that favor the null hypothesis are represented as BF_01_. A BF value of 3 or less is considered ambiguous evidence, and a BF value of 10 or greater indicates strong evidence.

## Results

### Percent correct

#### Shape-matching performance compared with chance (*t*-tests)

One-sample *t*-tests were conducted for each participant group to compare performance on each shape-matching task relative to chance (50%).

For the tactile–tactile task, the performance of each participant group was significantly higher than chance: Argus II participants, *t*(10) = 42.5, *p* < 0.001, *d* = 12.81, *N* = 11; sighted controls, *t*(9) = 38, *p* < 0.001, *d* = 12.01; sighted controls with simulated ultra-low vision, *t*(7) = 19.8, *p* < 0.001, *d* = 6.99 (Figure 3).

For the visual–visual task, the performance of each participant group was significantly higher than chance: Argus II participants, *t*(10) = 3.08, *p* = 0.012, *d* = 0.93, *N* = 11; sighted controls, *t*(9) = 37.99, *p* < 0.001, *d* = 12.01; sighted controls with simulated ultra-low vision, *t*(7) = 35, *p* < 0.001, *d* = 12.37 (Figure 3).

For the visual–tactile task, the performance of the Argus II participants was not significantly greater than chance, *t*(10) = 1.04, *p* = 0.325, *d* = 0.31, *N* = 11. The performance of both control groups on the visual–tactile task was significantly greater than chance: sighted controls, *t*(9) = 38.89, *p* < 0.001, *d* = 12.30; sighted controls with simulated ultra-low vision, *t*(7) = 31.84, *p* < 0.001, *d* = 11.26 (Figure 3).

#### Shape-matching group level comparison (ANOVAs)

The tactile–tactile task one-way ANOVA did not show a significant variation across the participant groups: Argus II participants (*M* = 0.98, *SD* = 0.04, *N* = 11); sighted controls (*M* = 0.97, *SD* = 0.04); sighted controls with simulated ultra-low vision (*M* = 0.94, *SD* = 0.06), *F*(2, 26) = 1.99, *p* = 0.157, $\eta_{p}^{2}$ = 0.13, BF_01_ = 1.36 (Figure 3).

The visual–visual task one-way ANOVA showed a significant variation across the participant groups: Argus II participants (*M* = 0.65, *SD* = 0.16, *N* = 11); sighted controls (*M* = 0.97, *SD* = 0.04); sighted controls with simulated ultra-low vision (*M* = 0.99, *SD* = 0.04), *F*(2, 26) = 32.12, *p* < 0.001, $\eta_{p}^{2}$ = 0.71, BF_10_ > 10 (Figure 3). When comparisons were made between the groups, the Argus II participants had significantly lower performance relative to the sighted controls and the sighted controls with simulated ultra-low vision: Argus II participants versus sighted controls, *t*(26) = 6.83, *p* < 0.001, *d* = 2.98; Argus II participants versus sighted controls with simulated ultra-low vision, *t*(26) = 6.81, *p* < 0.001, *d* = 3.17. The two control groups did not significantly differ in performance, *t*(26) = 0.39, *p* = 1.00, *d* = 0.18 (Figure 3).

The visual–tactile task one-way ANOVA showed a significant variation across the participant groups: Argus II participants (*M* = 0.57, *SD* = 0.23, *N* = 11); sighted controls (*M* = 0.98, *SD* = 0.04); sighted controls with simulated ultra-low vision (*M* = 0.97, *SD* = 0.04), *F*(2, 26) = 26.59, *p* < 0.001, $\eta_{p}^{2}$ = 0.67, BF_10_ >10 (Figure 3). When comparisons were made between the groups, the Argus II participants had significantly less accurate performance relative to the sighted controls and the sighted controls with simulated ultra-low vision: Argus II participants versus sighted controls, *t*(26) = 6.47, *p* < 0.001, *d* = 2.83; Argus II participants versus sighted controls with simulated ultra-low vision, *t*(26) = 5.90, *p* < 0.001, *d* = 2.74). The two control groups did not significantly differ in performance, *t*(26) = 0.18, *p* = 1.00, *d* = 0.09 (Figure 3).

### Correlation analyses

Overall, the performance of Argus II participants on the visual–visual and on the visual–tactile tasks were positively correlated with one another, *r*(9) = 0.84, *p* = 0.001, BF_10_ > 10, *N* = 11. The correlation between visual–tactile task performance and participant age was not significant, *r*(9) = 0.16, *p* = 0.65, BF_01_ = 2.46, *N* = 11. The correlation between visual–visual task performance and participant age was not significant, *r*(9) = 0.33, *p* = 0.32, BF_01_ = 1.73.

**Figure 4.**
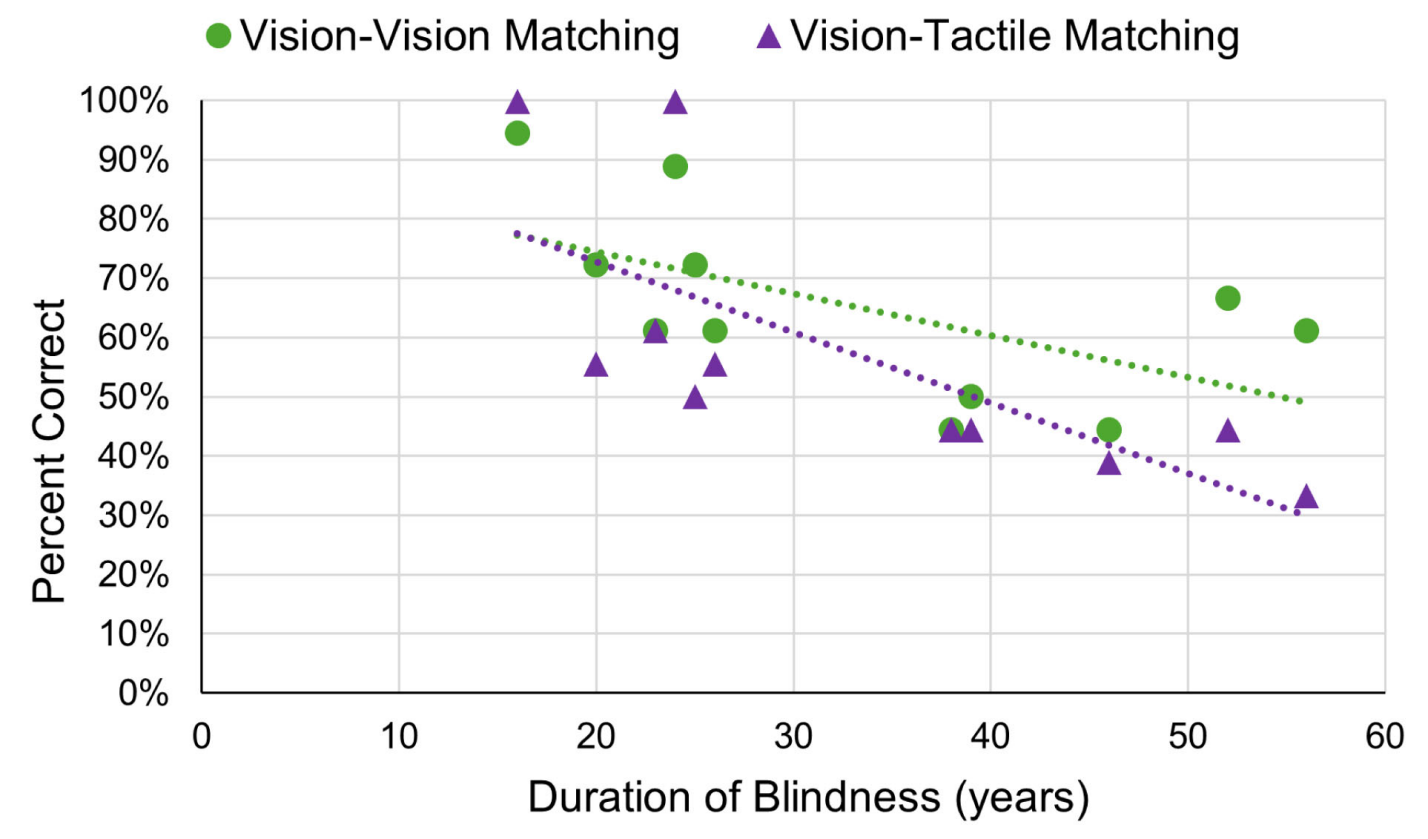
Shape-matching performance relative to blindness duration. Percent correct for visual–visual matching and visual–tactile matching in Argus II participants relative to the duration of blindness in years. Linear fits of the visual–tactile and visual–visual matching are shown as dotted lines in the color matching the relevant data points. Chance is 0.50 fraction correct (2AFC) for all of the shape-matching tasks. The duration of blindness is presented in years.

There was a significant negative correlation between visual–tactile task performance and duration of blindness (including the period of ultra-low Argus II vision in years), *r*(9) = –0.72, *p* = 0.012, BF_10_ = 5.87 (Figure 4). The negative correlation between performance on the visual–visual task and duration of blindness was not significant, *r*(9) = –0.59, *p* = 0.055, BF_01_ = 0.53 (Figure 4). However, based on BF, evidence for the null hypothesis is weak, suggesting that the correlation was trending toward significance.

Furthermore, to confirm the impact of prolonged blindness before implantation, we calculated the duration of blindness, not including the period of ultra-low Argus II vision. To do so, we subtracted the duration of Argus II use from the duration of blindness. The correlational trends remained the same. There was a significant negative correlation between the duration of blindness before implantation and visual–tactile task performance, *r*(9) = –0.67, *p* = 0.03, BF_10_ = 3.41. The negative correlation between the duration of blindness before implantation and visual–visual task performance was not significant, *r*(9) = –0.55, *p* = 0.08, BF_01_ = 0.70. However, the BF suggests that the correlation was trending toward significance.

The linear correlations between visual–visual and visual–tactile task performance and duration of prosthesis use were both not significant: visual–visual, *r*(9) = 0.19, *p* = 0.58, BF_01_ = 2.35; visual–tactile, *r*(9) = 0.23, *p* = 0.50, BF_01_ = 2.21. Upon visual inspection of the data, we decided to use a median split (median = 42 months) method when analyzing the correlation between task performances and the duration of prosthesis use to attempt to capture the correlational trends.

**Figure 5.**
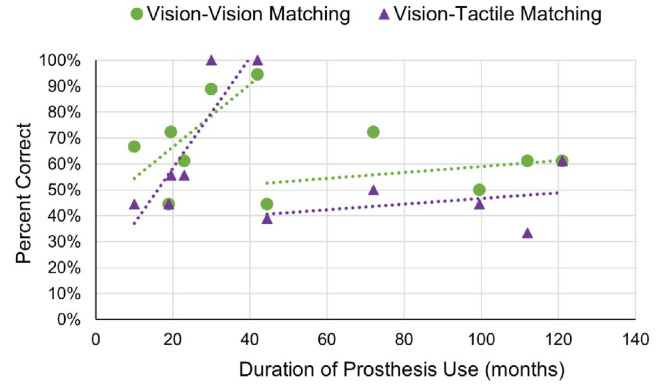
Shape-matching performance relative to duration of Argus II use. Percent correct for visual–visual matching and visual–tactile matching in Argus II participants relative to the duration of prosthesis use in months. Each participant is represented by two data points: green and purple (*N* = 11). Linear fits of the visual–tactile and visual–visual matching are shown as dotted lines in the color matching the relevant data points. Chance is 0.50 fraction correct (2AFC) for all of the shape-matching tasks. The duration of prosthesis use is presented in months.

Participants that had been using the Argus II prosthesis for 42 months or less (*n* = 6) showed a significant positive correlation between performance on the visual–tactile task and duration of prosthesis use, *r*(4) = 0.89, *p* = 0.018, BF_10_ = 4.62 (Figure 5). Although the positive correlation between visual–visual task performance and duration of prosthesis use was not significant, the BF indicates weak evidence for the null hypothesis, *r*(4) = 0.72, *p* = 0.11, BF_01_ = 0.68. However, based on the small BF, evidence for the null hypothesis is weak, suggesting that the correlation was trending toward significance. The data suggest that the participants’ performance increased in the early stages of prosthesis use.

Participants who had been using the prosthesis for more than 42 months (*n* = 5) did not show a significant correlation between visual–tactile performance and duration of use, *r*(3) = 0.32, *p* = 0.602, BF_01_ = 1.66 (Figure 5). The same was true for the correlation between visual–visual task performance and duration of prosthesis use, *r*(3) = 0.33, *p* = 0.59, BF_01_ = 1.64. The relatively small BFs for both correlations suggest that the evidence for the null hypothesis is weak.

### The *d*′ statistic

**Figure 6.**
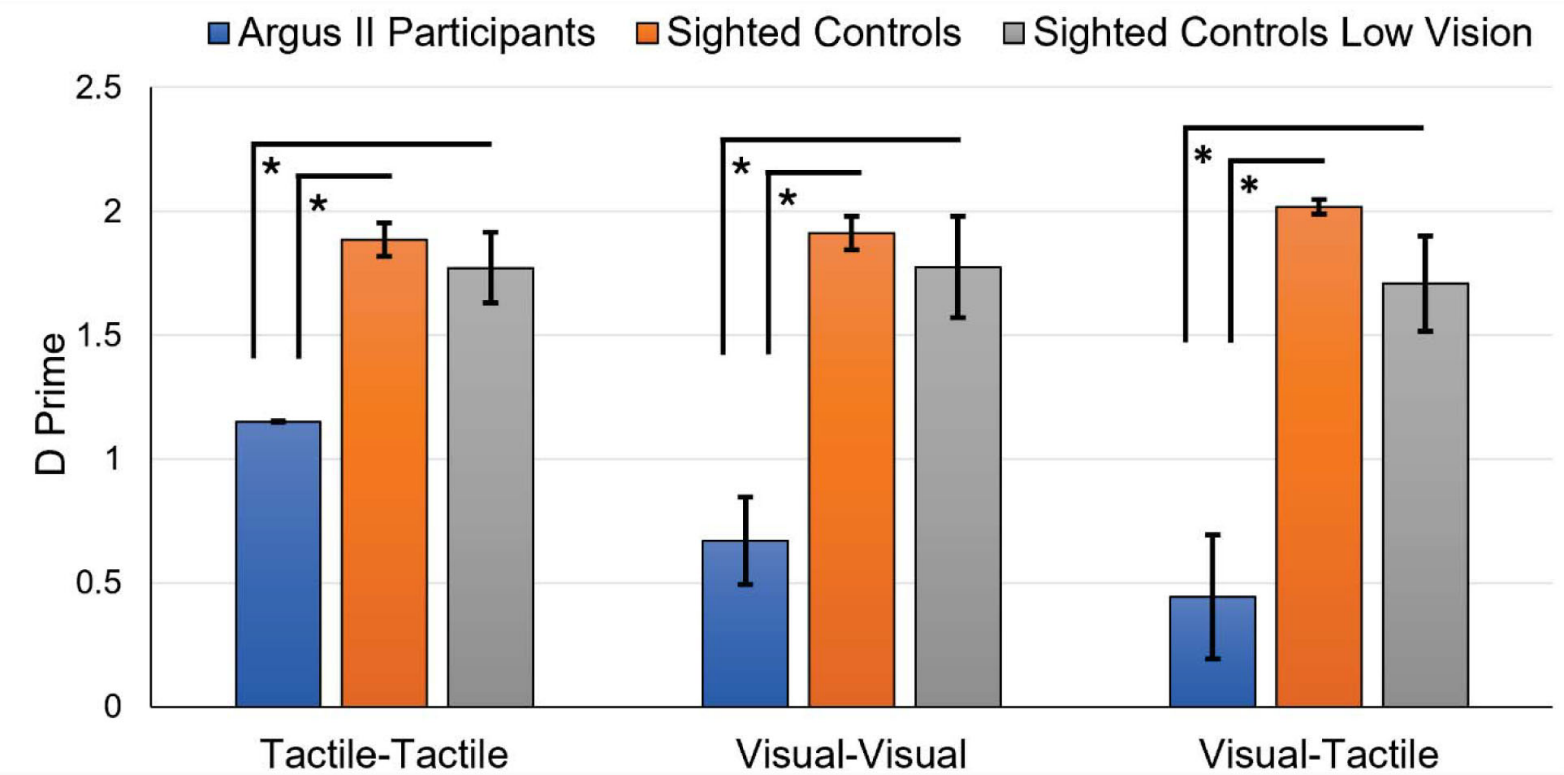
Shape task sensitivity measure (*d*′). Average sensitivity, as measured by *d*′, for the shape-matching tasks, comparing the Argus II participant group to the sighted participant group and the sighted participant group with simulated ultra-low vision. Error bars represent standard error.

Higher values of *d*′ indicate better discrimination or a larger separation between signal and noise. A *d*′ value of 0 indicates the inability to distinguish between signal and noise. The tactile–tactile task one-way ANOVA showed a significant variation in *d*′ scores across the participant groups: Argus II participants (*M* = 1.15, *SD* = 0.006, *N* = 11); sighted controls (*M* = 1.89, *SD* = 0.22); and sighted controls with simulated ultra-low vision (*M* = 1.77, *SD* = 0.40), *F*(2, 26) = 27.1, *p* < 0.001, $\eta_{p}^{2}$ = 0.68, BF_10_ > 10 (Figure 6). Comparisons between groups revealed lower *d*′ values for the Argus II participants compared with sighted controls and sighted controls with simulated ultra-low vision: Argus II participants versus sighted controls, *t*(26) = 6.86, *p* < 0.001, *d* = 3.00; Argus II participants versus sighted controls with simulated ultra-low vision, *t*(26) = 5.45, *p* < 0.001, *d* = 2.53. The two control groups did not significantly differ in *d*′ scores, *t*(26) = 0.98, *p* = 1.00, *d* = 0.46.

The visual–visual task one-way ANOVA revealed a significant variation in *d*′ scores across the participant groups: Argus II participants (*M* = 0.67, *SD* = 0.58, *N* = 11); sighted controls (*M* = 1.91, *SD* = 0.22); sighted controls with simulated ultra-low vision (*M* = 1.78, *SD* = 0.58), *F*(2, 26) = 20.2, *p* < 0.001, $\eta_{p}^{2}$ = 0.61, BF_10_ > 10 (Figure 6). Comparisons between groups revealed significantly lower *d*′ values for Argus II participants relative to sighted controls and sighted controls with simulated ultra-low vision: Argus II participants versus sighted controls, *t*(26) = 5.83, *p* < 0.001, *d* = 2.55; Argus II participants versus sighted controls with simulated ultra-low vision, *t*(26) = 4.87, *p* < 0.001, *d* = 2.26. The two control groups did not significantly differ in *d*′ values, *t*(26) = 0.59, *p* = 1.00, *d* = 0.28.

The visual–tactile task one-way ANOVA revealed significant variations in *d*′ across participant groups: Argus II participants (*M* = 0.44, *SD* = 0.83, *N* = 11); sighted controls (*M* = 2.02, *SD* = 0.09); sighted controls with simulated ultra-low vision (*M* = 1.71, *SD* = 0.55), *F*(2, 26) = 20.8, *p* < 0.001, $\eta_{p}^{2}$ = 0.62, BF_10_ > 10 (Figure 6). When comparisons were made, Argus II participants showed significantly lower *d*′ scores compared with sighted controls and sighted controls with simulated ultra-low vision: Argus II participants versus sighted controls, *t*(26) = 6.09, *p* < 0.001, *d* = 2.66; Argus II participants versus sighted controls with simulated ultra-low vision, *t*(26) = 4.60, *p* < 0.001, *d* = 2.14. Control groups did not significantly differentiate between their *d*′ scores, *t*(26) = 1.10, *p* = 0.84, *d* = 0.52.

### Response bias

A positive C value indicates a bias toward responding “different,” and a negative value indicates a “same” response (Macmillan & Creelman, 2005). A value of 0 indicates no bias in response.

**Figure 7.**
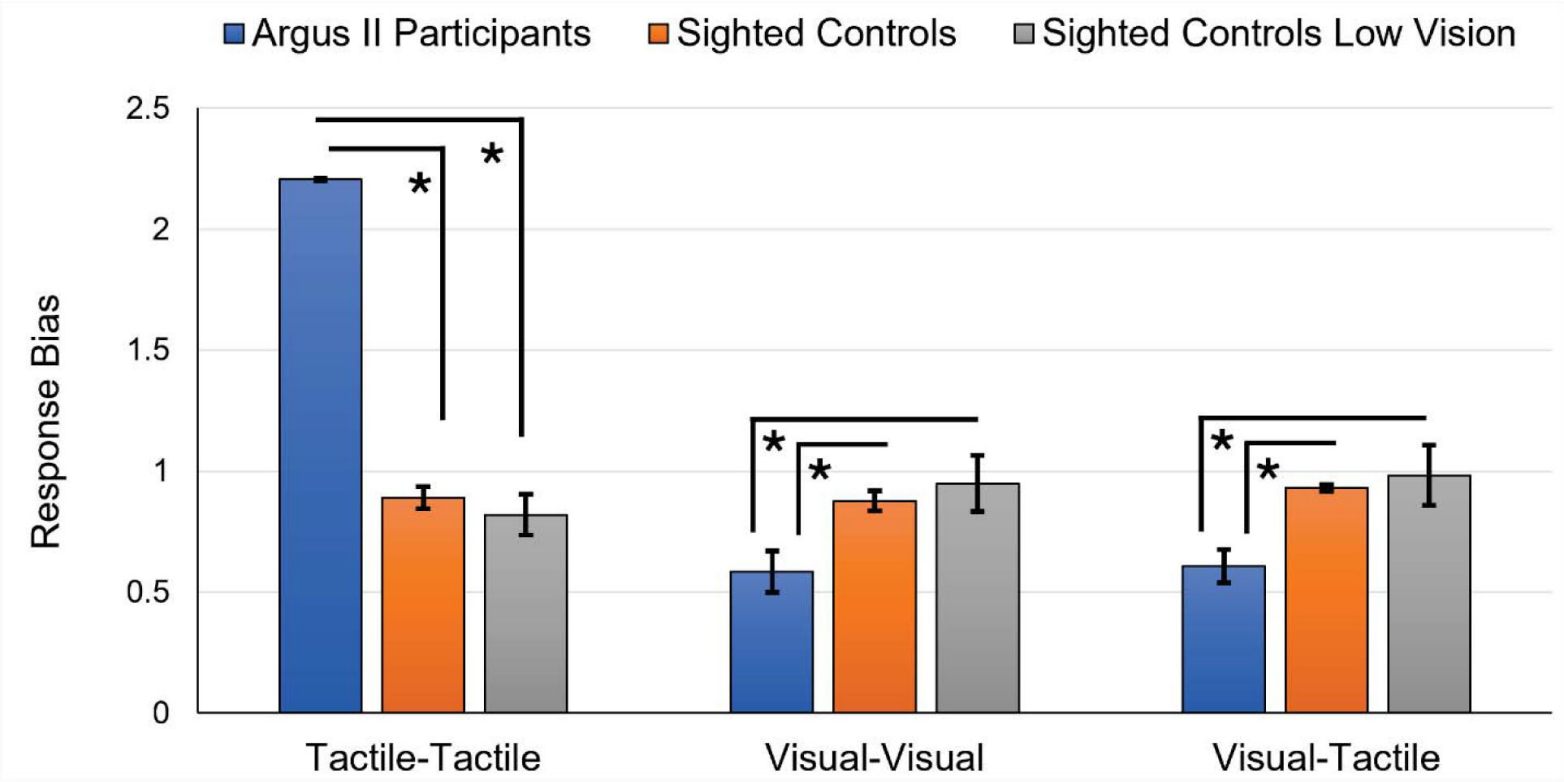
Shape task response bias. Average response bias for the shape-matching task for the Argus II participant group compared with the sighted participant group and the sighted participant group with simulated ultra-low vision. Higher values represent a tendency to respond “different” instead of “same” when comparing the two shapes presented in a trial. Error bars represent standard error.

The tactile–tactile task one-way ANOVA revealed significant differences in response bias across participant groups: Argus II participants (*M* = 2.21, *SD* = 0.02, *N* = 11); sighted controls (*M* = 0.89, *SD* = 0.15); sighted controls with simulated ultra-low vision (*M* = 0.82, *SD* = 0.24), *F*(2, 26) = 269.00, *p* < 0.001, $\eta_{p}^{2}$ = 0.95, BF_10_ > 10 (Figure 7). Comparisons among groups revealed that Argus II participants had significantly higher response bias values compared with the sighted control and sighted control with simulated ultra-low vision groups: Argus II participants versus sighted controls, *t*(26) = 19.82, *p* < 0.001, *d* = 8.66; Argus II participants versus sighted controls with simulated ultra-low vision, *t*(26) = 19.63, *p* < 0.001, *d* = 9.12. There was no difference in response bias between control groups, *t*(26) = 0.97, *p* = 1.00, *d* = 0.46.

The visual–visual task one-way ANOVA revealed significant differences in response bias between participant groups: Argus II participants (*M* = 0.58, *SD* = 0.28, *N* = 11); sighted controls (*M* = 0.88, *SD* = 0.13); sighted controls with simulated ultra-low vision (*M* = 0.95, *SD* = 0.33), *F*(2, 26) = 5.66, *p* = 0.009, $\eta_{p}^{2}$ = 0.30, BF_10_ = 6.19 (Figure 7). Comparisons revealed that Argus II participants had significantly lower response bias compared with sighted controls and sighted controls with simulated ultra-low vision: Argus II participants versus sighted controls, *t*(26) = 2.62, *p* = 0.04, *d* = 1.14; Argus II participants versus sighted controls with simulated ultra-low vision, *t*(26) = 3.06, *p* = 0.015, *d* = 1.42. There was no significant difference in response bias between control groups, *t*(26) = 0.59, *p* = 1.00, *d* = 0.28.

The visual–tactile task one-way ANOVA revealed significant differences in response bias between participant groups: Argus II participants (*M* = 0.61, *SD* = 0.23); sighted controls (*M* = 0.93, *SD* = 0.05); sighted controls with simulated ultra-low vision (*M* = 0.98, *SD* = 0.35), *F*(2, 26) = 7.90, *p* = 0.002, $\eta_{p}^{2}$ = 0.39, BF_10_ > 10 (Figure 7). Comparisons among groups revealed significantly lower response bias for Argus II participants compared with sighted controls and sighted controls with simulated ultra-low vision: Argus II participants versus sighted controls, *t*(26) = 3.27, *p* = 0.009, *d* = 1.43; Argus II participants versus sighted controls with simulated ultra-low vision, *t*(26) = 3.45, *p* = 0.006, *d* = 1.67. There was no significant difference between the control groups, *t*(26) = 0.48, *p* = 1.00, *d* = 0.24.

### Post-experiment survey

After completing the experiment, the Argus II participants were contacted and asked about their perceptual experience since receiving the Argus II prosthesis. For the original six Argus II participants, the survey question was asked approximately 5 years after completion of the experiment. Therefore, the average duration of prosthesis use for all participants at the time of the survey was 86.5 months. We received responses from 10 out of 11 participants. The most common response (*n* = 4) was that perceptual functionality remained unchanged over time. However, three participants stated that functionality consistently improved, and two reported a consistent decline. Finally, one participant reported a nonlinear effect.

**Figure 8.**
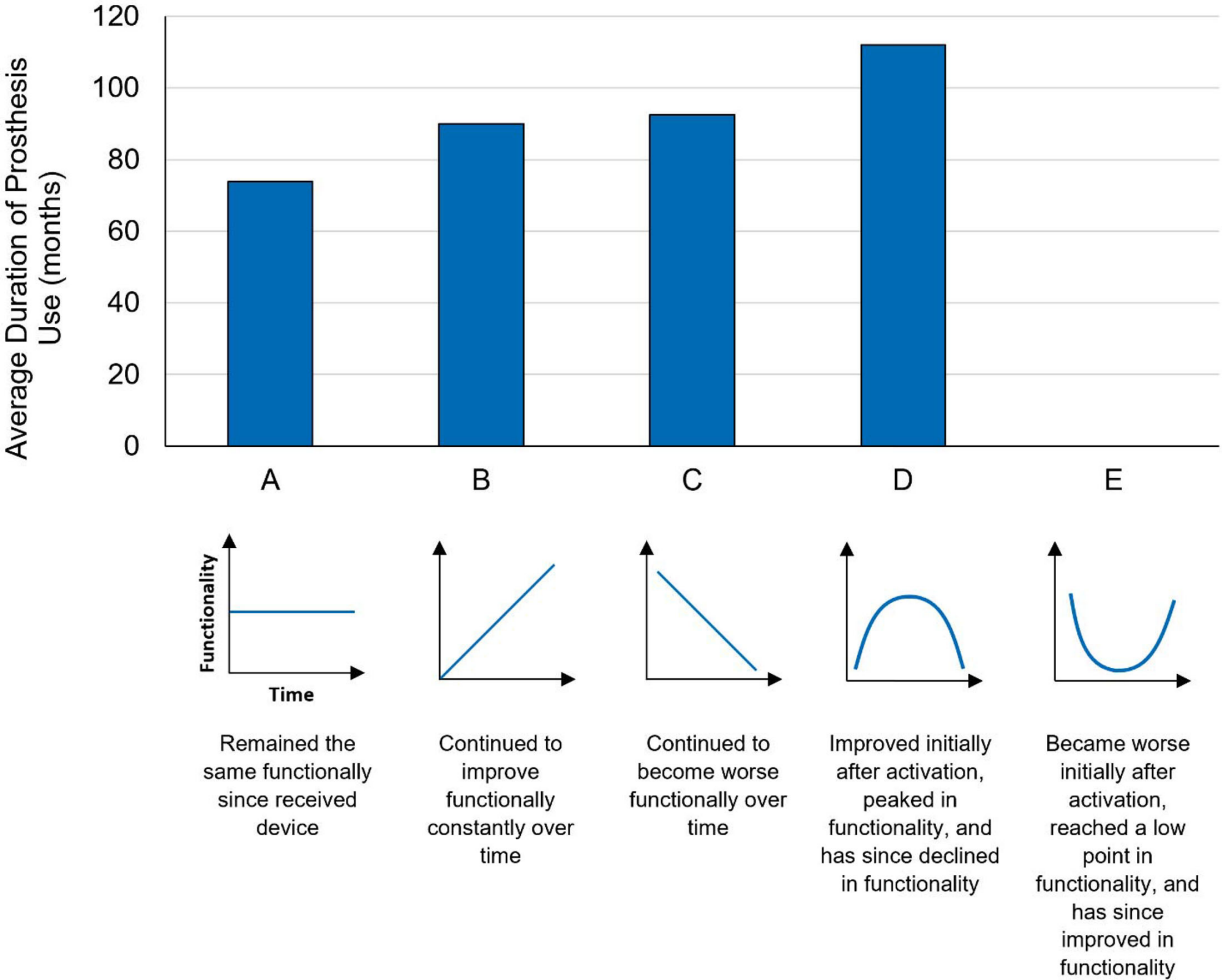
Argus II participant survey responses (*n* = 10). The average duration of prosthesis use (at the time of the survey) separated by survey response. A schematic of the performance over time is shown beneath each potential answer, in addition to the verbal description of the Argus II performance over time. The schematics are included only for explanatory purposes; the question was asked verbally with the description provided, and none of the schematics was shown to participants.

If participants are grouped based on their total duration of use (participant group divided equally into either below or above 80 months of use; see details in the Methods section) (Figure 8), all of the reports of consistent decline (option C) or improvement and then decline (option D) were reported by the longest users. Therefore, some of the bias toward no improvement or consistent improvement in the overall results may be due to differences in the duration of device use, with shorter users reporting more stable or improving function and longer users reporting more declining or variable function.

## Discussion

### Overview

Our previous paper investigated the ability of Argus II participants to integrate visual–tactile shape information and the interaction of their unisensory versus multisensory learning trajectories (Stiles et al., 2022). The current study extended our previous work by including a larger cohort of participants (*N* = 11) with greater variability in experience with the device. This allowed a closer examination of how multisensory performance changes over longer durations of device usage.

Argus II participants (*N* = 11) displayed the ability to learn unisensory shape matching, as evidenced by their above-chance performance on both the visual and tactile tasks. Due to the large variability in participant experience and performance, Argus II participants’ multisensory performance as a group (visual–tactile task) was not significantly above chance. The sighted control group and the simulated ultra-low-vision control group performed better than Argus II participants on the visual–visual and visual–tactile tasks.

The larger Argus II participant cohort also allowed us to explore the correlation between the duration of blindness and participant functionality with higher statistical power. We found that the longer duration of blindness negatively correlated with participant outcomes (even while controlling for participant age), indicating the potential limiting factor of atrophy and reorganization in participant outcomes.

We incorporated a signal detection analysis in the current study to determine differences in task strategy between Argus II participants and the sighted control groups. This is particularly important given that signal detection theory has not been applied to multisensory perception in retinal prosthesis users. We found significant differences between the Argus II participant cohort (*N* = 11) and the two control groups for both the measure of sensitivity and bias. These results have interesting implications for participant task strategy, which appeared to differ in the visually restored relative to sighted controls. Overall, the signal detection analyses confirmed that participants with the Argus II retinal prosthesis approached the shape-matching tasks with unique strategies.

### Performance with prolonged device use

Research involving visual restoration and retinal prostheses is relatively modern; therefore, few studies have examined functionality over extended periods of time. In our previous study with a smaller cohort of six participants (Stiles et al., 2022), the Argus II participants’ multisensory shape-matching performance (visual–tactile) was positively correlated with the duration of prosthesis use (up to 42 months). In this follow-up study, this correlation was no longer significant when additional participants (*n* = 5) with extended device experience (up to 121 months) were included. However, there was a significant positive correlation up to the median duration of use (42 months), followed by a positive (nonsignificant) slope beyond the median duration of use. These intriguing results may indicate that visual and multisensory performance improves the most during the initial stages of device usage and stagnates with prolonged device usage. To further evaluate performance over time, participants were asked in a follow-up survey about their perception of Argus II prosthesis performance over time. This follow-up question was asked approximately 5 years after the experiment completion for the six original participants. Therefore, 5 years was added to the duration of device use for these participants when analyzing survey responses (average 85.6 months). Of the 10 respondents, the shorter using participants reported mostly improvement or stable function, whereas the longer using participants also reported periods of decline in function.

For participants with an initial improvement in functionality, this may have been driven by their brain plastically adapting to vision or perceptual learning to improve task performance. Brain readaptation to vision at the cortical level has been supported by neuroimaging research of Argus II participants. Nadvar et al. (2022) showed that resting state connectivity between tactile and visual regions is altered in blindness and then restored to sighted dynamics with visual restoration. In addition, Stiles, Choupan, Ameri, Patel, and Shi (2025) found that visual cortical thickness, which atrophies during blindness, is partially rejuvenated with Argus II visual restoration. This research suggests that the brain is plastically readapting to the restored vision, which could be manifested in conscious or subconscious changes in visual perception. Participants will also likely utilize perceptual learning and develop techniques to better interpret artificial vision over time (Beyeler, Rokem, Boynton, & Fine, 2017). These techniques may include optimizing head movement to scan the environment and then integrating the temporally extended information into a cohesive single percept. In addition, stimuli are perceived as arcuate flashes of light (Beyeler et al., 2019; Erickson-Davis & Korzybska, 2021). Therefore, participants need to learn to ignore the shape of the phosphenes and their flashing dynamics (i.e., high spatial and temporal frequency information) and interpret the blurred holistic image over time (low-frequency information). This type of image interpretation is not automatic, as we tend to focus on edges and flashes (Tsuchiya & Koch, 2005), and likely requires training to perform. Therefore, it is likely that participants’ initial performance would improve based on these adjustments in strategy and perceptual learning of stimuli interpretation.

Argus II functionality could decline with longer device use for behavioral or physiological reasons. On the behavioral side, the Argus II device is tiring to use and requires extensive attention for minimal improvements in visual perception (Luo, Zhong, Clemo, & da Cruz, 2016; Luo & da Cruz, 2016; Zhou et al., 2013). Therefore, participants’ initial enthusiasm for using the device may wane over time. If participants use the device less frequently 5 or more years after implantation, this infrequent practice could diminish their functionality with the device. Alternatively, on the physiological side, the Argus II retinal prosthesis is a stimulation device implanted within the eye, and the electrode–tissue interface is altered after prolonged usage (Gregori et al., 2018). The device has been shown in some instances to move on the retinal surface (rotating around the single tack that keeps it in place) (Delyfer et al., 2018), and thresholds for electrodes have also been found to increase over time (Yue, Wuyyuru, Gonzalez-Calle, Dorn, & Humayun, 2020). Additionally, ongoing physiological remodeling of the retina, including synaptic reorganization, altered neuronal responsiveness, and changes in retinal structure such as thinning and displacement of inner retinal layers, may contribute to a gradual decline in Argus II functionality over time as the retina adapts to electrical stimulation and experiences progressive degeneration (Ashkenazy, Zann, & Gregori, 2020; Güven, Düzgün, Kutucu, & Gül, 2023; Patelli, Colombo, Aly, & Rossetti, 2021; Yue, Weiland, Roska, & Humayun, 2016). Both challenges with prolonged use could cause decreases in participant performance.

Another potential interpretation of our results is that two of the participants, who were high performers, were outliers and were randomly tested at the middle time point. However, the survey of participants’ self-report of functionality does not support this conclusion, as the longer using participants (>80 months) were more likely to report a period of decline in performance relative to the shorter using participants (<80 months).

Overall, our findings are limited by evaluating different participants over time rather than a longitudinal study of an individual participant's functionality at multiple time points. However, the benefit of the multi-participant, multi-timepoint design is that there is no shape task learning due to repeated sessions of the same object discrimination. At each data point, the participant perceived the stimuli for the first time. Future studies would add to our understanding of participant performance with the Argus II retinal prosthesis by evaluating each participant's functionality over time through several experimental sessions, particularly if a control task could be designed to filter out task learning. Conversely, future research could explore the impact of multimodal training—particularly visual–tactile—on enhancing visual performance. A clinical trial found that an eight-session rehabilitation program focusing on contrast sensitivity, object identification, visual scanning, and object tracking significantly improved functional vision and mobility in Argus II participants (National Institutes of Health, 2021). Because tactile training is a key component of rehabilitation for Argus II participants, directly examining how multimodal training influences visual improvement would be especially valuable.

Deafness rehabilitation with cochlear implants has been achieved in a larger participant population over a longer duration and, therefore, is an interesting comparison for this study of visual restoration with retinal prostheses (a relatively newer technology). Participants with cochlear implants who were investigated longitudinally also had a variety of participant outcomes (Tyler, Parkinson, Woodworth, Lowder, & Gantz, 1997). On average, participants improved in performance immediately following implantation (9 months) and then plateaued over time (maximum of 54 months). However, a subset of participants either showed no improvement over time or improved and then declined in their performance. These results mirror our study by indicating an early learning effect and individual differences in longer term performance trends.

Overall, the data in this paper support the hypothesis that Argus II participant functionality initially improves and then could plateau or decline with time. Prior research with Argus II participants highlighted multiple reasons for this potential pattern of functionality due to retinal, cortical, and cognitive changes. However, our results also highlight robust individual differences among participants. Further research with longitudinal studies and comparing data from multiple techniques (retinal imaging, brain imaging, and behavior) may better explain these differences.

### Impact of prolonged blindness on participant outcomes

In this study, we found a significant negative correlation between the duration of blindness and the visual–tactile matching performance of the Argus II participants. The correlation between the duration of blindness and visual–visual matching was also negative. Although it was not significant, it was trending toward significance based on the Bayes factor. Blindness increasingly impacts the neural networks and physiology of the retina and brain with longer durations, which could have a deleterious effect on visual restoration and generate this negative correlation.

Neuroimaging studies in late-blind participants with retinitis pigmentosa (the primary retinal disease of Argus II participants) have shown that visual pathways have transsynaptic degeneration (Wallerian degeneration) from the deteriorated retina propagating up to the visual cortex (Rita Machado et al., 2017). In addition, magnetic resonance imaging studies have found that visual regions are reorganized during late blindness to process remaining sensory modalities, among other tasks (Amedi et al., 2007; Sadato, Okada, Kubota, & Yonekura, 2004). Finally, at the retinal level, prolonged blindness from retinitis pigmentosa has been found to reorganize the neural network and significantly alter the functionality of retinal neurons (Ji, Zhu, Grzywacz, & Lee, 2012; Jones et al., 2016). Therefore, it is not surprising that longer periods of blindness (and presumably increased atrophy and reorganization) could have heightened adverse effects on participant outcomes with visual restoration.

A parallel form of sensory restoration with a biomedical implant is auditory rehabilitation with cochlear implants (mentioned above). Cochlear implants have also been investigated to determine whether the cortical changes during deafness impact auditory restoration. It was found that greater reorganization and atrophy deteriorated participant outcomes (Sandmann et al., 2012). It has also been shown that the period of deafness also negatively correlated with cochlear implant participant outcomes (Bernhard et al., 2021), as we have seen with the Argus II retinal prosthesis participants in this study. Although both children and adults benefit from cochlear implants, recent trends emphasize pediatric implantation to leverage the advantages of early auditory stimulation. Overall, this research highlights the importance of implanting participants shortly after visual impairment to ensure the best rehabilitation outcomes.

### Signal detection analysis

The *d*′ scores were used as a measure of sensitivity, with higher values indicating a better ability to discriminate between signal and noise. Argus II participants displayed significantly lower sensitivity than both sighted control groups across all shape-matching tasks. In other words, Argus II participants were worse at discriminating between signal and noise during shape-matching tasks. Although the percent correct analysis indicated significant differences between the visual–visual and visual–tactile tasks between the Argus II participants and sighted controls, the more sensitive measure of *d*′ also (surprisingly) revealed significant differences between groups for the tactile–tactile task, as well. The differences in tactile–tactile task performance indicate that group differences are not just perceptual in nature (as artificial vision and natural vision are quite different), but rather that there was variability in the approach to the shape task itself.

To further evaluate these differences in task approach, we investigated response bias (C). Whereas *d*’ considers both strategy and performance, response bias (C) focuses more on the individual's decision-making strategies. Response bias is used to determine a participant's bias toward answering “same” or “different” during a task, with higher values indicating a bias toward answering “different.” Notably, no participants in the current study (across all three groups) displayed a negative response bias score. Therefore, all participants in the current study tended to respond “different” rather than “same.” In both the visual–visual and visual–tactile tasks, Argus II participants were more hesitant to respond “different” than both sighted control groups. However, Argus II participants were abundantly more likely to respond “different” for the tactile–tactile task than both control groups.

This large difference in tactile–tactile bias may be due to the development of different tactile strategies during blindness. Blind individuals rely more heavily on non-visual senses, such as audition and somatosensation, to process information. This over-reliance may influence their decision-making strategies over time. For example, Dufour, Després, and Candas (2005) reported higher response biases and greater sensitivity towards auditory echo cues in blind individuals than in sighted controls. Additionally, sensitivity and response bias were reported to be higher in blind subjects compared with sighted controls during a tactile heat discrimination task (Slimani, Ptito, & Kupers, 2015). These results mirror the heightened response bias in the tactile–tactile shape-matching task in this paper in the Argus II participants relative to the sighted controls. This difference in tactile-task bias indicates that, despite visual restoration, the Argus II participants still performed more similarly to the blind than the sighted regarding task strategy in a tactile task. Alternatively, although tactile sensation is heightened in blindness and low vision, visual perception declines. Therefore, it is logical that the visual–visual and visual–tactile matching would have the opposite change in bias (i.e., a lower bias) for the weakened sense (vision) in the Argus II participants relative to the normally sighted.

In addition to differences in visual perception (i.e., artificial vision), Argus II participants may develop different decision-making strategies and biases during blindness. Overall, the signal detection analyses confirmed that participants with the Argus II retinal prosthesis approach shape-matching tasks with unique strategies.

We propose that these strategy differences should be evaluated in future studies to better understand the positive and negative effects of task strategy differences. For example, a strong bias toward “same” or “different” in an evenly divided task (half “same” answers and half “different” answers) can lead participants to perform worse than chance and may indicate a maladaptive approach to perceptual tasks and task learning.

## Conclusions

The current study examined visual and multisensory performance in a cohort of participants implanted with the Argus II retinal prosthesis. Notably, the Argus II participants had a wide range of experience with the device (∼10–120 months), which allowed us to investigate how prolonged device usage impacts performance. Although there was high variation between subjects, shape-matching performance (on average) seemed to improve during the initial stages of device use and potentially stagnate or decline in performance over time. We also found that Argus II participants with longer durations of blindness performed worse at matching shapes across the senses. Additionally, a signal detection analysis confirmed that participants implanted with the Argus II device showed differences in shape-matching strategies compared with sighted controls. The current study is a key step toward better understanding how visual and multisensory performance is impacted by artificial vision and how performance changes throughout the course of device use.

## Supplementary Material

Supplement 1
