# Supplementary material for: Visual–tactile shape perception in Argus II Participants: The impact of prolonged device use and blindness on performance
Source: J Vis. 2025 Oct 14;25(12):19. doi: 10.1167/jov.25.12.19 (PMC12530443; doi:10.1167/jov.25.12.19)
Supplement: Supplement 1 [file jovi-25-12-19_s001.docx]

**Supplemental Figures and Tables**

Visual-Tactile Shape Perception in the Visually Restored with Artificial Vision


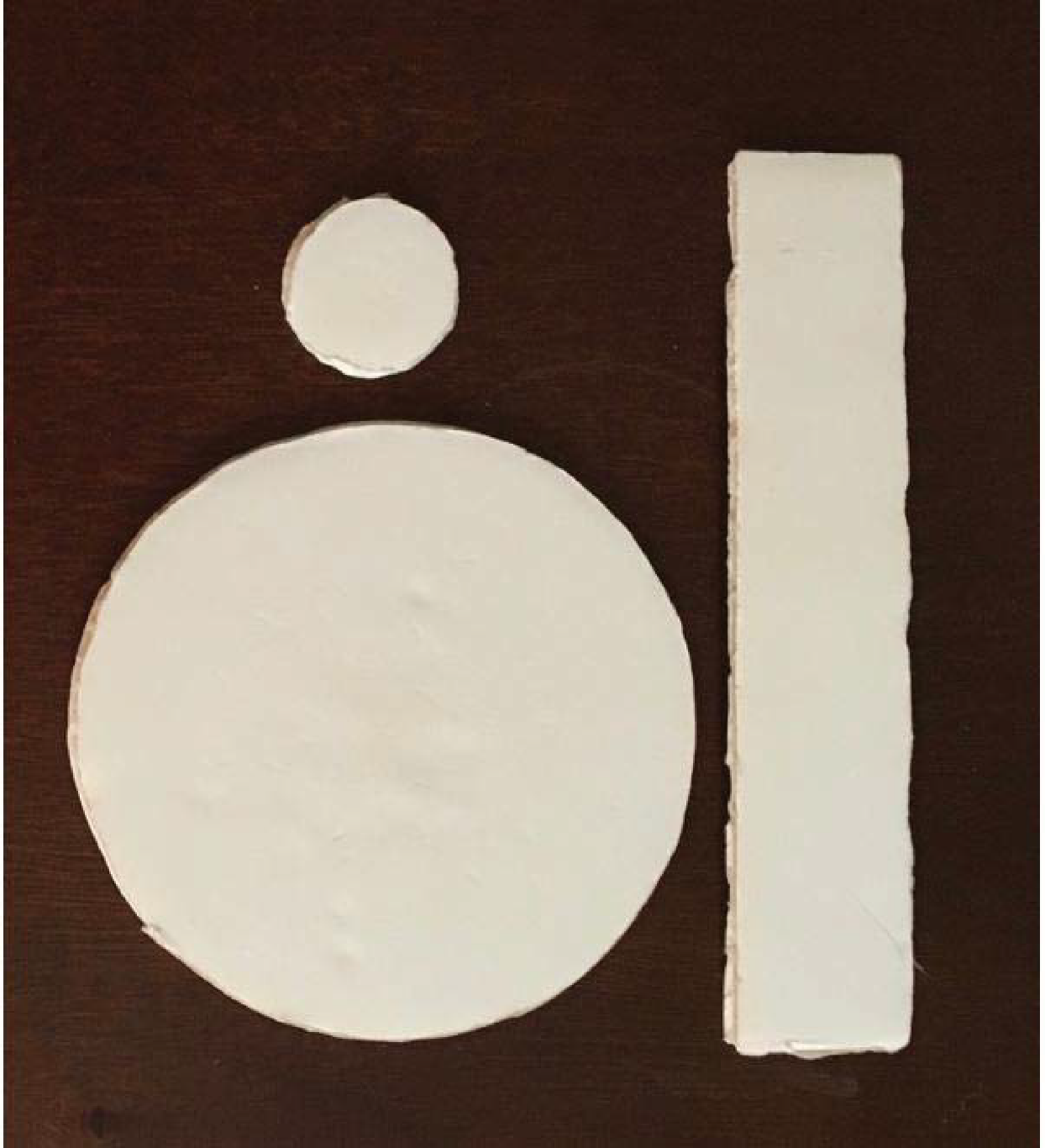


**Figure S1: Image of the Experiment Shapes.** The image presents the three physical shapes used in the visual and tactile tasks. The large circle is shape 3, and the small circle is shape 4. The vertical rectangular shape served as shape 1 (horizontal rectangle) and shape 2 (vertical rectangle). Figure 3A presents a schematic of the shapes used. The image presents the shapes on a dark wood table; the experiments were performed on a black felt-covered table.


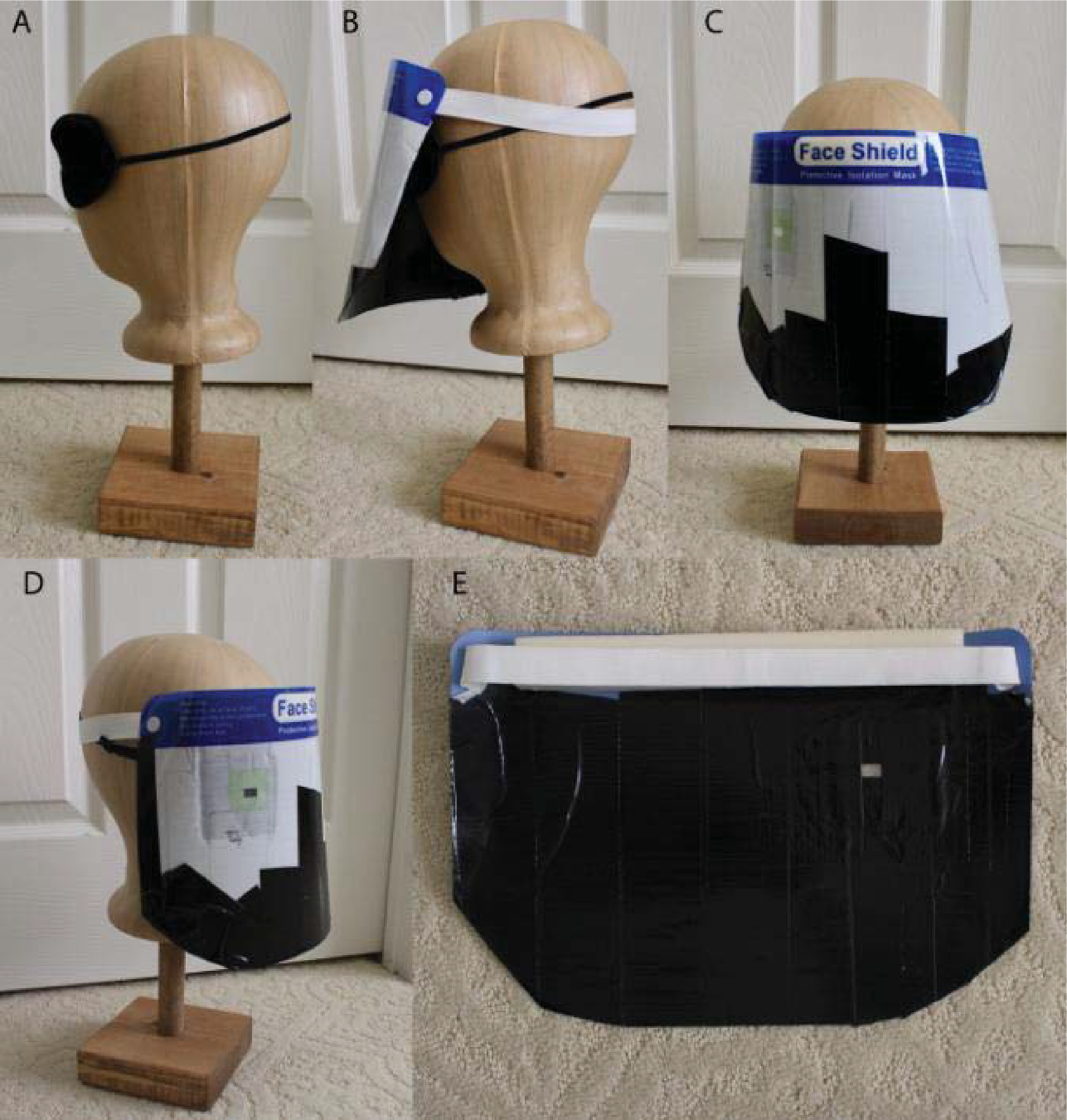


**Figure S2: Images of the Eye Patch and Opaque Face Mask for Sighted Controls.** Image A shows the eye patch used for occluding the left eye during the experiment. Image B shows the opaque face mask placed over the eye patch on the left eye (configuration for experiment). Images C and D show the front of the opaque face mask with all the vision from the right eye blocked except a small rectangle (which has 4 blurring films layered over the opening). Finally, Image E presents the inside of the opaque face mask (the view of the participant) and the small window for the right eye to view the environment.

| **Subject ID** | **Age (years)** | **Gender** | **Glasses or Contacts** | **Hearing Loss Left Ear** | **Hearing Loss Right Ear** |
| --- | --- | --- | --- | --- | --- |
| **S1** | 61 | M | Yes | None | None |
| **S2** | 66 | F | Yes | None | None |
| **S3** | 68 | F | Yes | None | None |
| **S4** | 69 | F | Yes | None | None |
| **S5** | 64 | F | No | Mod | Mod |
| **S6** | 58 | F | Yes | None | None |
| **S7** | 63 | F | Yes | None | None |
| **S8** | 69 | F | Yes | None | None |
| **S9** | 55 | M | Yes | None | None |
| **S10** | 62 | M | No | None | None |

*M = Male, F = Female, None = No Hearing Loss, Mild = Mild Hearing Loss,*

*Mod = Moderate Hearing Loss, and Sev = Severe Hearing Loss*

**Table S1: Sighted Controls Information.** Sighted controls’ age, gender, visual aid use (glasses or contacts), and hearing impairment are listed in Table S1. All information is self-reported by the participants at the time of the experiment.

| **Subject ID** | **Age (years)** | **Gender** | **Right Eye Acuity** | **Right Eye Acuity with Mask** |
| --- | --- | --- | --- | --- |
| **SLV1** | 48 | F | 20/60+4 | 20/600 |
| **SLV2** | 35 | M | 20/20 | 20/600+2 |
| **SLV3** | 61 | F | 20/25+3 | 20/800+4 |
| **SLV4** | 31 | M | 20/40+4 | 20/600+4 |
| **SLV5** | 68 | F | 20/80+4 | 20/1000-1 |
| **SLV6** | 33 | M | 20/40+4 | 20/600+4 |
| **SLV7** | 25 | F | 20/20 | 20/1000-2 |
| **SLV8** | 34 | F | 20/50+4 | 20/1000+3 |

*SVL = Simulated Low Vision, M = Male, F = Female*

**Table S2: Sighted Controls with Simulated Ultra-Low Vision Information.** Sighted controls’ age, gender, and visual acuity are listed in Table S2. The subjects are numbered with the abbreviation SLV, Simulated Low Vision. The age and gender information are self-reported by the participants at the time of the experiment. The right eye acuity was measured with an eye patch over the left eye. The right eye acuity with the mask was measured with the eye patch on the left eye and an opaque face mask covering the face and right eye (Figure S2). The numbers indicate how much of that line was reported and/or correct. A positive number indicates which of the reported letters were correct. A negative number indicates how many letters were missed when only a majority of a line was legible. Details on the procedure for measuring visual acuity are in the Methods Section.

| **Subject ID** | **Participated in rehabilitation training?** | **Total rehabilitation training hours** | **Did the patient visit a university or Second Sight for additional testing? *** | **Frequency of Argus II home use** |
| --- | --- | --- | --- | --- |
| **A1** | Yes | 12 Hours | Yes, visited Second Sight several times for 1-week visits | Once per day |
| **A2** | Yes | 1 Hour | Yes, visited USC about 10 times for 1-day visits, visited Second Sight about 10 times for 1-day visits | Once per day |
| **A3** | Yes | 24 Hours | Yes, visited Second Sight once | Every other day |
| **A4** | Yes | 30 Hours | Yes, visited Second Sight five times for 1-week visits | Once per week |
| **A5** | No | N/A | Yes, visited USC over 5 times for 1-day visits | 3-4 times per week |
| **A6** | Yes | 13 Hours | Yes, visited University of Minnesota four times for 1-2 days each | Every day |
| **A7** | Yes | 150 Hours | No | Once per day |
| **A8** | No | N/A | Yes, visited for a total of 165.5 hours | Once a week |
| **A9** | No | N/A | Yes, for a total of 48 hours | Once a month |
| **A10** | No | N/A | Yes, for a total of 225 hours | Once per day |
| **A11** | Yes | 14 Hours | No | Once a month |

**This excludes university visit(s) for this study*

**Table S3. Argus II Training Information.** The Argus II patients self-reported the directed and informal training they have received with the Argus II visual prosthesis. If the Argus II patient participated in the Second Sight administered rehabilitation training, column two is reported as a “Yes”. The total duration of the Second Sight rehabilitation training, as estimated by the patient is reported in column two of the table. Visits to universities for research studies can also act like training, especially if feedback is given during the experimental tasks. All the study visits to universities or Second Sight Medical Products as reported by patients are detailed in column 4 of the table. The final table column lists the frequency of the informal device use in the patient’s home.
